# Separation of Poly(styrene-*block-t*-butyl methacrylate) Copolymers by Various Liquid Chromatography Techniques

**DOI:** 10.1100/2012/932609

**Published:** 2012-03-12

**Authors:** Tina Šmigovec Ljubič, David Pahovnik, Majda Žigon, Ema Žagar

**Affiliations:** ^1^Laboratory for Polymer Chemistry and Technology, National Institute of Chemistry, Hajdrihova 19, 1000 Ljubljana, Slovenia; ^2^Center of Excellence for Polymer Materials and Technologies, Tehnološki Park 24, 1000 Ljubljana, Slovenia; ^3^University College for Polymer Technology, Glavni trg 1, 2380 Slovenj Gradec, Slovenia; ^4^EN-FIST Centre of Excellence, Dunajska 156, 1000, Ljubljana, Slovenia

## Abstract

The separation of a mixture of three poly(styrene-*block-t*-butyl methacrylate) copolymers (PS-*b*-P*t*BMA), consisting of polystyrene (PS) blocks of similar length and *t*-butyl methacrylate (P*t*BMA) blocks of different lengths, was performed using various chromatographic techniques, that is, a gradient liquid chromatography on reversed-phase (C18 and C8) and normal-phase columns, a liquid chromatography under critical conditions for polystyrene as well as a fully automated two-dimensional liquid chromatography that separates block copolymers by chemical composition in the first dimension and by molar mass in the second dimension. The results show that a partial separation of the mixture of PS-*b*-P*t*BMA copolymers can be achieved only by gradient liquid chromatography on reversed-phase columns. The coelution of the two block copolymers is ascribed to a much shorter P*t*BMA block length, compared to the PS block, as well as a small difference in the length of the P*t*BMA block in two of these copolymers, which was confirmed by SEC-MALS and NMR spectroscopy.

## 1. Introduction

Over the past few decades block copolymers have attracted significant scientific and economic interest since they are able to self-assemble into long-range-ordered structures in bulk, while in selective solvents they form micellar aggregates with a core-shell structure in which the insoluble block forms the core and the soluble block forms the surrounding corona. Stability and an ability to vary the core-shell dimensions make block copolymers widely applicable in various fields, for example, for colloidal stabilization, the compatibilization of polymer blends, controlled drug delivery, water purification, gene therapy, phase-transfer catalysis, viscosity, and surface modifications [1]. Block copolymers can show a distribution in more than one property, that is, chemical composition, functionality, architecture, and molar mass, and as such they are considered as complex polymers. By using conventional methods of polymer characterization, such as IR or NMR, only the average structural features of copolymers without their distributions can be determined [2]. The chemical composition distribution (CCD) and functionality distribution of copolymers can be determined by liquid chromatography (LC) based on different mechanisms of separation, for example, liquid-adsorption chromatography (LAC), which is a gradient method and liquid chromatography under critical conditions (LCCC), which is an isocratic method [3]. LAC separation is directed by enthalpic interactions, while in LCCC the enthalpic interactions are compensated by entropy losses of the solute [4].

 The molar-mass averages and distributions of polymers are usually determined using size-exclusion chromatography (SEC), which is governed by entropic effects and results in the elution of macromolecules according to their hydrodynamic volume. SEC in combination with different detectors (e.g., UV/VIS, IR) also gives information about the chemical composition as a function of the molar mass. However, only the average composition at a certain molecular size can be determined. For high-throughput screening and analysis, short and wide-bore columns were developed, making a high-speed separation possible [5].

 By combining different LC techniques (LAC or LCCC with SEC) into a two-dimensional chromatographic system, it is possible to correlate a certain composition (or functionality) with the molar mass [6, 7]. The optimal analysis time in two-dimensional liquid chromatography (2D-LC) is achieved if the second dimension is as short as possible, while giving sufficient resolution, which is fulfilled by wide-bore high-speed SEC columns. The complete transfer of fractions between both dimensions is possible using a software-controlled automatic valve equipped with two sample loops. Automatization of the 2D-LC system has resulted in numerous applications of 2D separations [8–11].

Living polymerization techniques (e.g., anionic polymerization) allow the synthesis of well-defined polymers. For the synthesis of functional (co)polymers, protected monomers with masked functional groups have been employed. For example, amphiphilic poly(styrene-*block*-methacrylic acid) copolymers (PS-*b*-PMAA) have been prepared by the deprotection of the protected carboxylic groups of poly(styrene-*block*-*t*-butyl methacrylate) copolymers (PS-*b*-P*t*BMA). The PS-*b*-PMAA can self-assemble into polymeric micelles and/or vesicles with nanometre-sized dimensions while being dissolved in a selective solvent for one of the blocks—typically water. These copolymers have been employed as materials in semiconductors, nanoparticles, micelle-encapsulated carbon nanotubes, and so forth. However, since all application fields require a well-defined structure and molar mass of the PS-*b*-PMAA copolymers various 2D-LC techniques have been developed for their simultaneous determination [12].

The diblock PS-*b*-P*t*BMA copolymers were widely studied by Glöckner et al. [13, 14] using gradient liquid chromatography on different stationary phases, for example, bare silica gel, phenyl, and octadecyl columns. On the silica gel column with the isooctane/THF mobile phase the separation of PS-*b*-P*t*BMA copolymers was not efficient, while it was possible to separate admixed PS from the copolymer. In contrast, the separation of PS-*b*-P*t*BMA copolymers was successful on phenyl or octadecyl stationary phases with methanol (MeOH)/THF gradients. The retention volume decreased with increasing P*t*BMA content in the block copolymer. This was in contrast to the elution of PS-*b*-PMMA in the gradient elution on a silica stationary phase where the retention was increased by increasing the content of the PMMA in the copolymer, since this block is more polar than the P*t*BMA block. The inversed elution order was explained by the bulky *t*-butyl group, which shields the polar ester group.

Zimina et al. [15] characterized PS-*b*-P*t*BMA using LCCC. Under the critical conditions for the P*t*BMA block they were able to examine the length of the PS block in the block copolymers by using different binary solvent mixtures and different PS standards. Critical conditions for the P*t*BMA block were established in acetonitrile/dichloromethane and THF/dichloromethane.

 Raust et al. [16] developed a 2D-LC method for complex (meth)acrylate-based copolymers synthesized by free-radical copolymerization, that is, isobutyl acrylate, isobutyl methacrylate, isobornyl acrylate, isobornyl methacrylate, and 2-etylhexyl acrylate. In the first dimension the CCD of the copolymers was determined using gradient chromatography on a reversed-phase column in the acetonitrile/THF mobile phase and in the second dimension the molar-mass characteristics of the fractions eluted from the first dimension were determined by fast SEC in THF.

 Falkenhagen et al. [17] determined the lengths of both blocks in PMMA-*b*-P*t*BMA copolymers using reversed-phase and normal-phase LCCC.

The aim of our work was to chromatographically evaluate a mixture of three linear PS-*b*-P*t*BMA copolymers consisting of polystyrene (PS) and poly(*t*-butyl methacrylate) (P*t*BMA) blocks. The PS-*b*-P*t*BMA copolymers were synthesized using living anionic polymerization and are well defined with respect to their molecular and structural characteristics. For a powerful 2D-LC characterization of the PS-*b*-P*t*BMA mixture a sufficiently high resolution in the first dimension is essential. Therefore, much of our work was dedicated to a determination of the chemical composition distribution using different liquid-chromatographic techniques (gradient LC and LCCC) and different stationary phases (C18 and C8 reversed phase and SiO_2_ normal phase). The optimized chromatographic conditions in gradient liquid chromatography were established by studying the elution behaviour of the PS and P*t*BMA homopolymers under different gradients. For the LCCC measurements a series of PS homopolymers of different molar masses and uniform molar-mass distributions were tested in different mobile phase compositions. Both LC methods were finally hyphenated with high-speed SEC in the second dimension.

## 2. Experimental Methods

### 2.1. Materials

#### 2.1.1. Block Copolymers

The PS-*b*-P*t*BMA copolymers were synthesized in the laboratory of Professor Dr. Nikos Hadjichristidis, Industrial Chemistry Laboratory, Polymers Group, University of Athens [18]. The copolymers are of low-molar-mass dispersity and with a defined length of both blocks. The number average molar mass of the PS block is about 29500 g/mol in all the samples, whereas that of the P*t*BMA block increases in the order copolymer 1 (3000 g/mol) < copolymer 2 (7000 g/mol) < copolymer 3 (32000 g/mol).

#### 2.1.2. Solvents

The solvents used for the gradient LC on the reversed-phase column were acetonitrile (ACN gradient grade; Sigma Aldrich, Germany) and tetrahydrofuran (THF p.a.; Merck, Germany). The solvents used for the gradient LC on the normal-phase column were *n*-hexane (p.a.; Merck, Germany) and THF. All the solvents were filtered through a Millipore filter system (0.45 *μ*m, Nylon 66, Supelco) and degassed with a Brason 2210 ultrasonic degasser.

### 2.2. NMR

The ^1^H NMR spectra of the PS-*b*-P*t*BMA samples were recorded on a 300-MHz Unity Inova 300 spectrometer (Varian) in the pulse Fourier Transform mode with a relaxation delay of 5 s and an acquisition time of 3 s. Tetramethylsilane (Me_4_Si, *δ* = 0) was used as an internal chemical-shift standard. The PS-*b*-P*t*BMA copolymers were dissolved in CDCl_3_-*d*_1_ as a solvent. Trifluoroacetic acid was added to solutions of block copolymers in order to shift the peak of the water in chloroform towards a lower magnetic field. The spectra were recorded immediately after the addition of the acid to avoid the hydrolysis of the *t*-butyl ester groups.

### 2.3. Size Exclusion Chromatography Coupled to a Multiangle Light Scattering Photometer (SEC-MALS)

The SEC-MALS measurements were performed at room temperature using a Hewlett-Packard pump series 1100 coupled to a DAWN HELEOS laser photometer with a GaAs linearly polarized laser (*λ*_0_ = 658 nm) and to an Optilab rEX interferometric refractometer (RI) operating at the same wavelength as the photometer (both instruments are from Wyatt Technology Corp., USA). The separations were carried out using a 5 *μ*m Resipore SDV column with a precolumn (300 mm length and 7.5 mm i.d., Polymer Laboratories) in THF. The Resipore SDV column exhibits a wide pore-size distribution, covering the molar masses from 200 to 400,000 Da. The nominal eluent flow rate was 1.0 mL/min. The mass of the samples injected onto the column was typically 3 × 10^−4^ g, whereas the solution concentration was 3 × 10^−3^ g/mL. The calculation of the molar-mass averages from MALS requires a sample-specific refractive-index increment (*dn*/*dc*), which was determined from the known weight fractions of the PS and P*t*BMA blocks, which were determined based on proton NMR results (Table 1) and known refractive-index increments of the corresponding homopolymers in THF (0.184 mL/g for PS and 0.065 mL/g for P*t*BMA). For the data acquisition and evaluation the Astra 5.3.4 software (Wyatt Technology Corp., USA) was utilized.

### 2.4. HPLC

#### 2.4.1. Gradient LC × SEC

The retention behaviour on reversed-phase (C18 and C8) columns was studied using a series of narrow-molar-mass dispersity P*t*BMA standards (10,000 and 30,000 g/mol) and PS standards (10,300 and 29,510 g/mol) at a solution concentration of 2 mg/mL in THF. On the normal-phase column the retention behaviour was studied using a series of PS standards (29,510 and 50,000 g/mol) and P*t*BMA standards (10,000 and 30,000 g/mol) at a solution concentration of 2 mg/mL in THF. A sample mixture was prepared by mixing stock solutions of each block copolymer with a concentration of 2 mg/mL in THF in a volume ratio of 1 : 1 : 1.

For the reversed-phase gradient LC experiments LC Nucleosil C18 column (250 mm × 4 mm I.D., Macherey-Nagel GmbH) and Zorbax Eclipse XDB-C8 (150 mm × 4.6 mm I.D., Agilent Technologies) were used. For the normal-phase gradient LC experiments a Nucleosil-Si (250 mm × 4 mm I.D., Macherey-Nagel GmbH) was used. The flow rate in the first dimension was 0.5 mL/min, while in the 2D-LC experiments it was reduced to 0.04 mL/min. In the second dimension a PSS SDV high-speed column (50 mm × 20 mm I.D., Polymer Standards Service GmbH) was used, and the THF flow rate was set to 3.0 mL/min. The PSS high-speed SDV column exhibits a wide pore-size distribution, covering the molar masses from 500 to 60.000 Da. The SEC column was calibrated using polystyrene standards of a narrow-molar-mass distribution with a concentration of 1 mg/mL. For the 2D-LC experiments a Perkin Elmer series 200 and Agilent 1100 pumps and an evaporative light-scattering detector ELS 1000 (Polymer Laboratories) were used. For the data acquisition and evaluation a WinGPC v. 7 (Polymer Standards Service GmbH) was utilized. The fractions were transferred from the first to the second dimension using an eight-port transfer valve equipped with two 100 *μ*L sample loops (EHC8W type, VICI Valco Instruments) that was controlled by a 2D WinGPC v. 7 software module.

#### 2.4.2. LCCC × SEC

The critical conditions for PS on the normal-phase column were determined in the mobile phase *n*-hexane and THF at different volume ratios by injecting the PS standards of 2,100 g/mol, 51,000 g/mol, and 97,200 g/mol at a solution concentration of 2 mg/mL THF. In these experiments we used a UV DAD Perkin Elmer series 200 as a detector at *λ* = 254 nm. In the 2D LCCC×SEC experiments the flow rate in the first dimension was reduced to 0.1 mL/min, while the THF flow rate in the second dimension was 4.0 mL/min.

## 3. Results and Discussion

### 3.1. Composition of PS-*b*-P*t*BMA Copolymers

**Figure 1:**
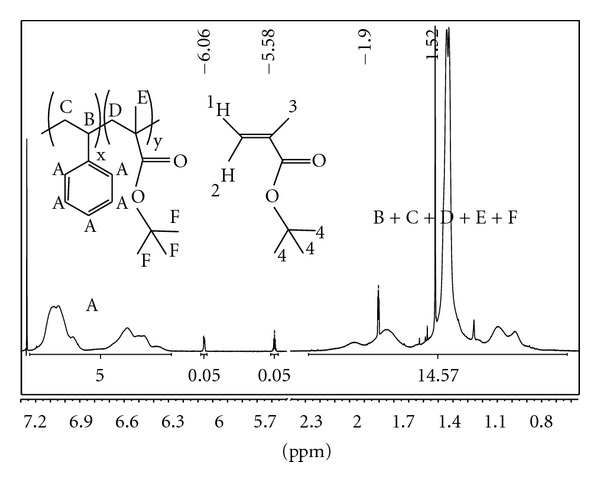
^1^H NMR spectrum of the PS-*b*-P*t*BMA copolymer number 3.

The molar ratio between the PS and P*t*BMA repeat units in the PS-*b*-P*t*BMA copolymers was determined using ^1^H NMR spectroscopy. The signals in the range from 7.24 ppm to 6.28 ppm correspond to the PS phenyl ring (A). The signals in the range from 2.38 ppm to 0.63 ppm belong to the PS-*b*-P*t*BMA backbone, that is, three protons to the PS backbone (methylene group C and methine group B) and two protons to the P*t*BMA backbone (methylene group D), and to the methyl (E) and *t*-butyl side groups (F) of the P*t*BMA block. The ^1^H NMR spectra of the PS-*b*-P*t*BMA samples denoted by 1 and 3 show the presence of minute amounts of residual *t*BMA monomer with the following signals: ^1^H NMR (CDCl_3_) *δ* (ppm) 6.06 (1H, dq, *J*_1,3_ = 1 Hz, *J*_1,2_ = 1.5 Hz), 5.58 (1H, dq, *J*_1,2_ = 1.5 Hz, *J*_2,3_ = 1.5 Hz), 1.90 (3H, dd, *J*_1,3_ = 1 Hz, *J*_2,3_ = 1.5 Hz), 1.52 (9H, s) (Figure 1).

The molar ratio between the PS and P*t*BMA repeat units was determined by comparing the integral of the PS aromatic protons in the between region 7.24–6.28 with the integral of the P*t*BMA protons, taking into account that at the equimolar ratio the ratio between them is 5 : 14. The integral for the P*t*BMA protons was obtained from the integral ranging from 0.63 to 2.38 ppm by subtraction of the integral for the protons of the PS backbone (C and D) and the integral of the protons of the methyl (4) and *t*-butyl (F) groups of the *t*BMA monomer. In the calculations we neglected the copolymers' terminal groups since the PS-*b*-P*t*BMA samples have high molar masses. The molar ratios between the PS and P*t*BMA repeat units in the copolymers were 1 : 0.067 for sample 1, 1 : 0.179 for sample 2, and 1 : 0.779 for sample 3.

From the molar ratios between the PS and P*t*BMA repeat units in the copolymers we calculated the corresponding weight fractions (*w*_PS_, *w*_P*t*BMA_), which were then used for the calculation of the copolymers' refractive-index increments (*dn*/*dc*, equation (1)). Consequently, these values were applied for the determination of the absolute molar-mass averages and the molar-mass distributions of the copolymers from the MALS photometer (Table 1):

(1) $$\begin{array}{c} \frac{dn}{dc}={\left(\frac{dn}{dc}\right)}_{\text{PS}}w_{\text{PS}}+{\left(\frac{dn}{dc}\right)}_{\text{P}t\text{BMA}}w_{\text{P}t\text{BMA}}, \end{array}$$

where (*dn*/*dc*)_PS_ and (*dn*/*dc*)_P*t*BMA_ are the refractive-index increments of the corresponding homopolymers in THF (0.184 mL/g for PS and 0.065 mL/g for P*t*BMA), while *w*_PS_ and *w*_P*t*BMA_ are the weight fractions of the PS and P*t*BMA repeat units in the copolymers, respectively.

### 3.2. Molar-Mass Averages and Molar-Mass Distribution of PS-*b*-P*t*BMA Copolymers

**Figure 2:**
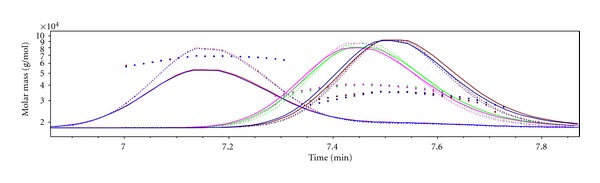
SEC-MALS chromatograms (the dashed lines indicate the DRI response, the dotted lines indicate the LS response at 90° angle) and molar mass versus elution volume curves for the PS-*b*-P*t*BMA copolymers. From left to right: copolymer 3, copolymer 2, and copolymer 1.

**Figure 3:**
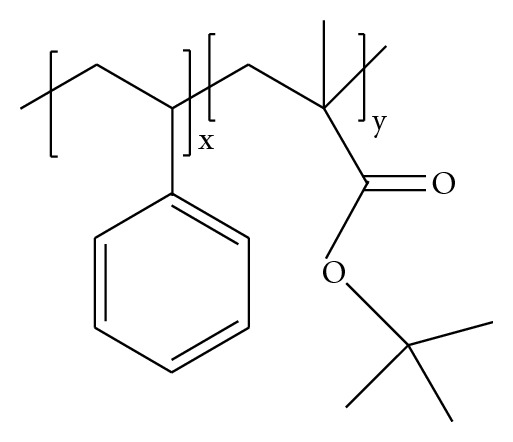
Schematic structure of PS-*b*-P*t*BMA; copolymer 1: *x* = 279, *y* = 14; copolymer 2: *x* = 290, *y* = 38; copolymer 3: *x* = 326, *y* = 187.

The SEC-MALS curves of the PS-*b*-P*t*BMA copolymers are symmetrical (Figure 2), and the calculated molar-mass dispersities (*D*_*M*_ ~ 1.03) indicate the very narrow-molar-mass distribution of the copolymers (Table 1). The molar-mass averages decrease in the order: copolymer 3 > copolymer 2 > copolymer 1. All three copolymers have comparable lengths of the PS block, while the length of the P*t*BMA block decreases in the same order as the copolymers' molar-mass averages (Table 1). The copolymers 1 and 2 have very short P*t*BMA blocks, when compared to that of the PS block. In addition, the difference in the P*t*BMA block length in these two copolymers is small (Table 1 and Figure 3).

### 3.3. Reversed-Phase Liquid Chromatography of PS-*b*-P*t*BMA Copolymers

**Figure 4:**
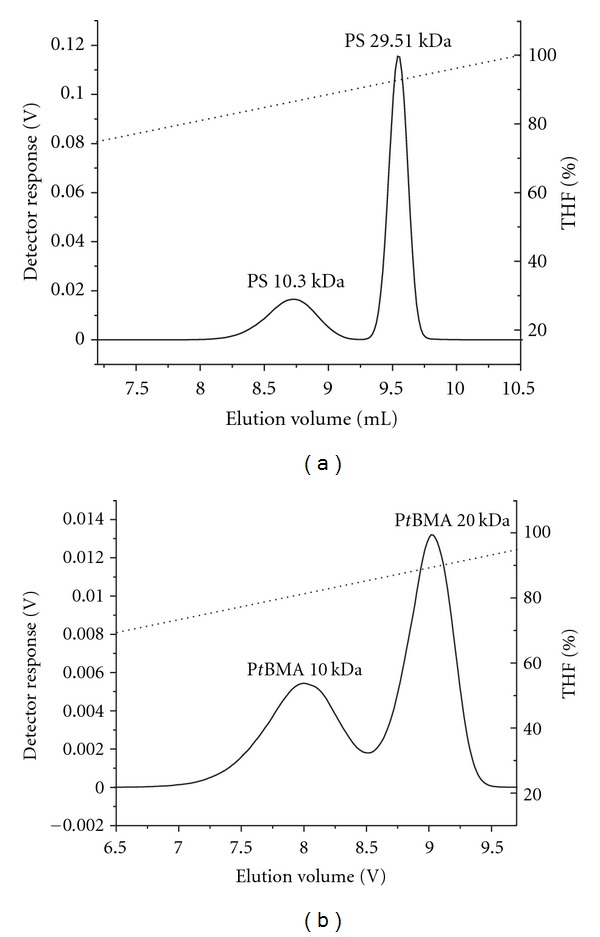
Gradient separation of PS (a) and P*t*BMA (b) homopolymers on the reversed-phase C18 column in the ACN/THF mobile phase at a flow rate of 1 mL/min.

The mixture of three copolymers was analysed using gradient HPLC on C18 and C8 reversed stationary phase columns, which is a frequently used chromatographic method for the separation of copolymers based on their chemical composition [19]. By changing the composition of the mobile phase during the run the solvent power of the mobile phase is adjusted to control the solubility and/or adsorption of the individual sample's constituents so that the CCD of the copolymers can be determined. In order to optimize the chromatographic conditions for the efficient separation of the PS-*b*-P*t*BMA copolymers, we first studied the elution behaviour of the individual homopolymers (PS and P*t*BMA) with a narrow-molar-mass distribution in the binary mobile phase, consisting of high- and medium-polarity solvents, that is, acetonitrile (ACN) and THF. Starting with the higher content of the ACN as a nonsolvent for homopolymers and gradually raising the amount of THF in the mobile phase as a thermodynamically good solvent for homopolymers, elution according to Martin's rule was achieved (Figures 4(a) and 4(b)). The homopolymer with a higher molar mass was eluted after the homopolymer with the lower mass. The elution volume of the P*t*BMA homopolymer was slightly lower than that of the PS homopolymer of comparable molar mass, due to the higher polarity of the P*t*BMA, compared to the PS homopolymer. Martin's rule correlates the retention factor with the number of repeat units in the homopolymer in a semiempirical relation. The retention factor (*k*′) increases exponentially with the number of homopolymer repeat units (*n*) according to the following

(2) $$\begin{array}{c} \ln k^{\prime }=\ln A+Bn, \end{array}$$

where *A* and *B* are empirical constants. The relation between ln⁡*k*′ and the chain length, that is, the number of repeat units, *n*, is linear [20].

**Figure 5:**
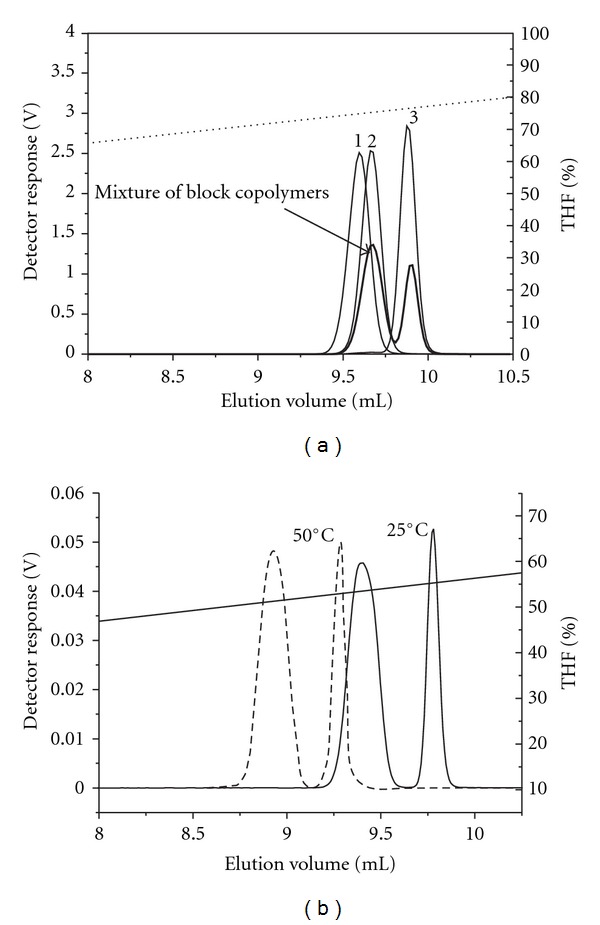
Overlay of the ELS curves of individual PS-*b*-P*t*BMA copolymers and their mixture (a) and overlay of ELS curves of the mixture of block copolymers obtained at 50°C and 25°C (b); gradient ACN/THF, flow rate: 0.5 mL/min, column: Nucleosil C18.

The overlay of chromatograms obtained with the gradient elution (ACN/THF) of the individual block copolymers and their mixture on the reversed-phase C18 column show that the peak eluted at a small elution volume in the chromatogram of the mixture belongs to the coeluted block copolymers 1 and 2, while the peak at a larger elution volume represents the block copolymer 3, which has the longest P*t*BMA block (Figure 5(a)).

Although we varied the chromatographic conditions by changing the gradient and the temperature of the measurements (Figure 5(b)) we were not able to separate the block copolymers 1 and 2 to the baseline, most probably due to the relatively short P*t*BMA block, when compared to the length of the PS block in the copolymers. At a higher temperature, that is, 50°C, the block copolymers eluted at slightly lower elution volumes, but without any improvement in resolution (Figure 5(b)). Glöckner and Wolf showed that the elution of the PS-*b*-P*t*BMA copolymers on the reversed-phase C18 column was governed mainly by the solubility/precipitation mechanism, while on the phenyl column it was the adsorption mechanism [14].

**Figure 6:**
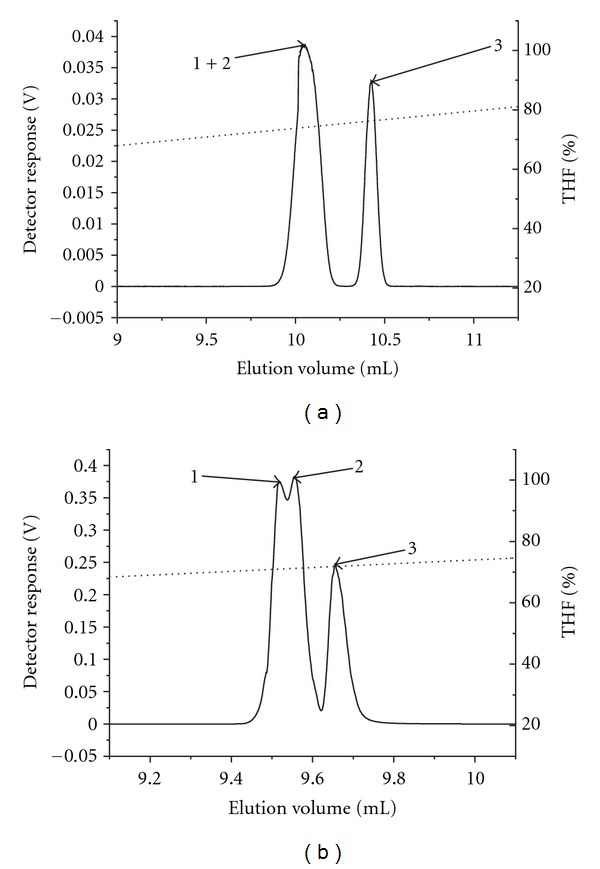
Gradient separation of the mixture of block copolymers on reversed-phase columns Nucleosil C18 (a) and Zorbax C8 (b) in ACN/THF at a flow rate of 0.04 mL/min.

The elution of the copolymers PS-*b*-P*t*BMA was also performed on the C8 stationary phase, since we expected a better resolution, as block copolymers differ only in the length of the P*t*BMA block, which is slightly more polar than the PS block (Figures 6(a) and 6(b)).

The PS-*b*-P*t*BMA copolymers elute from the C8 column somewhat earlier than from the C18 column since the former stationary phase has a slightly higher polarity than the latter. The resolution between the PS-*b*-P*t*BMA samples 1 and 2 slightly improved, while that between the samples 2 and 3 worsened, compared to the results obtained on the C18 column (Figures 6(a) and 6(b)).

### 3.4. Normal-Phase Liquid Chromatography of PS-*b*-P*t*BMA Copolymers

**Figure 7:**
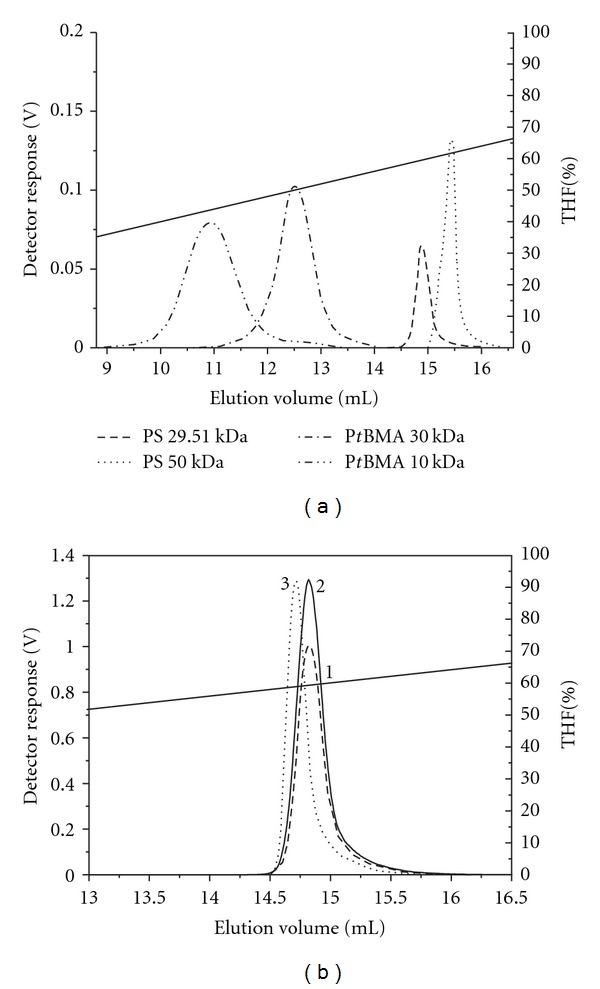
Overlay of ELS chromatograms of PS and P*t*BMA homopolymers (a) and individual block copolymers PS-*b*-P*t*BMA (b); gradient* n*-hexane/THF, flow rate 0.5 mL/min, column: Nucleosil Si.

For the normal-phase HPLC of the individual homopolymers and the PS-*b*-P*t*BMA copolymers we used the *n*-hexane/THF binary solvent gradient similar to the one used by Karanam et al. [21], who investigated the P*t*BMA-*b*-PMMA-*b*-P*t*BMA triblock copolymers. Since the polarity of the P*t*BMA is slightly higher than that of the PS we expected that the P*t*BMA homopolymers will elute on the normal-phase column at larger elution volumes than the PS homopolymers of comparable molar masses. Our results show the opposite elution order, that is, the P*t*BMA homopolymers eluted before the PS homopolymers (Figure 7(a)), which could be a consequence of the inaccessibility of the P*t*BMA ester carbonyl group for the interaction with the stationary phase due to the shielding effect of the bulky *t*-butyl side group. Overall, the results show that the separation of the copolymer mixture on the normal-phase column using the *n*-hexane/THF gradient (Figure 7(b)) is worse than that on the reversed-phase columns (Figure 6).

### 3.5. Liquid Chromatography of PS-*b*-P*t*BMA Copolymers under Critical Conditions

**Figure 8:**
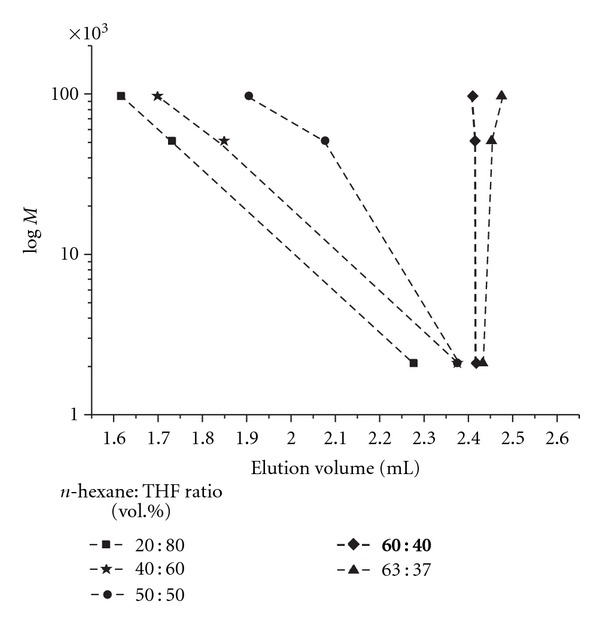
Determination of critical conditions for polystyrene on normal phase Nucleosil Si 300 $\overset{´}{\text{Å}}$ column using a different composition of *n*-hexane/THF mobile phase at a temperature of 55°C and a flow rate of 0.5 mL/min. UV detector UV (*λ* = 254 nm).

For block copolymers that are eluted under critical conditions for one block the other block defines the mode of elution, that is, the exclusion or adsorption mode. Under critical conditions for the PS block the elution of the PS-*b*-P*t*BMA copolymers is governed only by the molar mass of the P*t*BMA block. The critical conditions for PS on the normal-phase column were determined at 55°C by injecting PS standards of different molar masses using the *n*-hexane/THF mobile phase of different compositions. At the volume ratio of *n*-hexane/THF = 60/40 the PS homopolymers eluted at the same elution volume regardless of their molar mass (Figure 8), which is in accordance with the literature data [8].

**Figure 9:**
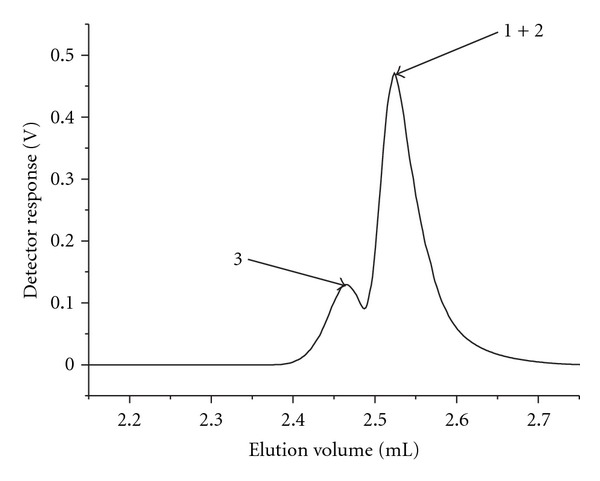
The chromatogram of the mixture of block copolymers obtained under critical conditions for polystyrene (*n*-hexane/THF: 60/40, *T* = 55°C) on normal-phase Nucleosil Si 300 $\overset{´}{\text{Å}}$ column at a flow rate of 0.5 mL/min. ELSD detector.

The separation of the mixture of PS-*b*-P*t*BMA copolymers under the critical conditions for the PS shows that the P*t*BMA block eluted under the exclusion mode (Figure 9). Unfortunately, the separation was less efficient than that using gradient LC on reversed-phase columns, which is most probably a consequence of the fact that the critical conditions for P*t*BMA (*n*-hexane/THF = 57 : 43) [17] are close to those for PS, which indicate not much difference in the polarity of both constituting blocks.

### 3.6. Two-Dimensional LC of PS-*b*-P*t*BMA Copolymers

Two-dimensional experiments on PS-*b*-P*t*BMA copolymers were performed only under HPLC conditions giving the best separation of copolymers according to composition, that is, in the first dimension we used liquid chromatography on the reversed-phase C18 and C8 columns. In the second dimension we used the high-speed SEC column, which separates the copolymers according to molar mass. A separate SEC separation of the mixture of block copolymers on this column results in one broad peak with a shoulder.

**Figure 10:**
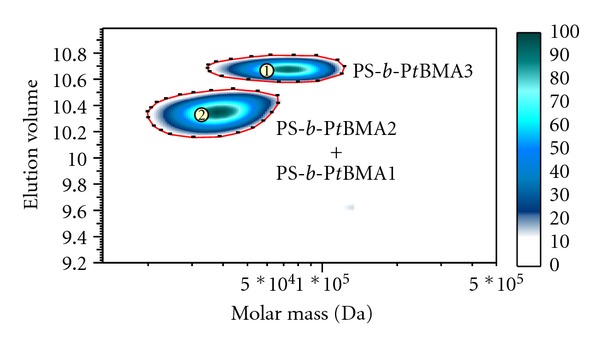
2D-LC separation of the mixture of block copolymers. 1D: Nucleosil C18 column, gradient ACN/THF, 0.04 mL/min. 2D: PSS SDV High-Speed column, THF, 3 mL/min. ELSD detector.

In 2D-LC, the 2D plot is presented as a contour plot in which the first dimension separation is represented along the *y*-axis (gradient LC, C18 or C8) and the second dimension (SEC) along the *x*-axis. The contour plot of the 2D-LC separation of the mixture of copolymers using the reversed-phase C18 column in the first dimension (Figure 10) shows two baseline separated spots, among which the spot denoted with 2 represents the coeluted block copolymers 1 and 2, whereas the spot denoted with 1 represents the block copolymer 3. Since in spot 2 two copolymers coeluted, it is broader along the *y*-axis in comparison with spot 1.

**Figure 11:**
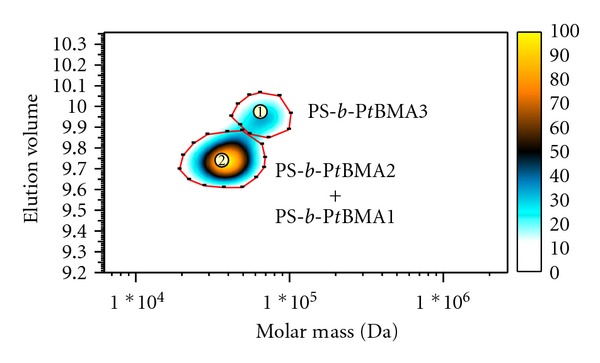
2D-LC separation of the mixture of block copolymers. 1D: Zorbax C8, gradient ACN/THF, 0.04 mL/min. 2D: PSS SDV High-Speed, THF, 3 mL/min. ELSD detector.

A similar 2D-LC elution pattern of the block copolymers was obtained when we used the C8 instead of the C18 column in the first dimension (Figure 11), although a one-dimensional gradient HPLC separation on the C8 column gave partially separated peaks for the block copolymers 1 and 2 (Figure 6(b)). In addition, the intensity of the spots in the 2D-LC contour plot is lower when using the C8 instead of the C18 column. This suggests that part of the sample is retained on the column, most probably due to the stronger interaction of the PS-*b*-P*t*BMA copolymers with the C8 rather than the C18 stationary phase, especially if the copolymer contains a longer P*t*BMA block (sample 3). Such interactions and the partial penetration of the solute into the narrow pores of the stationary phase, which result in a fully retained sample and a decreased sample recovery, were already reported in the literature [22, 23]. The results of the molar-mass determination in the 2D-LC experiments indicate that the relative molar-mass values of the PS-*b*-P*t*BMA copolymers are in relatively good agreement with the absolute ones and that spot two represents the average molar mass of the copolymers 1 and 2 (Table 2).

## 4. Conclusion

We studied the elution behaviour of a mixture of three PS-*b*-P*t*BMA copolymers using various liquid-chromatography techniques. The PS-*b*-P*t*BMA copolymers were of a defined structure with similar PS block lengths and different P*t*BMA block lengths. The composition of the mixture was studied by reversed-phase (C18 and C8) and normal-phase liquid chromatography and liquid chromatography under critical conditions for PS. The results showed that the most efficient separation of copolymers according to their composition was achieved using reversed-phase liquid chromatography. On the C8 column the resolution between the PS-*b*-P*t*BMA copolymers 1 and 2, which both consist of much shorter P*t*BMA block as compared to the length of PS block, slightly improved, while the resolution between the copolymer 2 and the copolymer 3, which contains much longer P*t*BMA block, worsened, compared to the results obtained on the C18 column. Since most promising separation according to copolymer composition was obtained by reversed-phase LC technique, it was used as a method for the composition separation of copolymers in the first dimension of 2D-LC experiments. The results of the 2D chromatographic separation revealed that none of the techniques used was able to completely separate the copolymer mixture, which was ascribed to the much shorter length of the P*t*BMA blocks, compared to the PS block in the two block copolymers of the mixture and the small difference in the P*t*BMA block length in these PS-*b*-P*t*BMA copolymers.

## Figures and Tables

**Table 1:** Molar-mass characteristics of the PS-*b*-P*t*BMA copolymers as determined by SEC-MALS.

Sample PS-*b*-P*t*BMA	*M*_*w*_ × 10^−3^ g/mol	*M*_*n*_ × 10^−3^ g/mol	*M*_*w*_/*M*_*n*_	^1^mol_PS_	^2^ *w*_PS_	^3^ *dn*/*dc* (mL/g)	*M*_*n*_ × 10^−3^ PS block g/mol	*M*_*n*_ × 10^−3^ P*t*BMA block g/mol
1	32.0	31.0	1.03	0.937	0.916	0.174	29.0	2.0
2	36.6	35.6	1.03	0.848	0.804	0.161	30.2	5.4
3	62.0	60.4	1.03	0.562	0.484	0.123	33.9	26.5

^1^mol_PS_: the mol fraction of PS was determined from proton NMR spectra.

^2^ *w*_PS_: the weight fraction of PS was determined from the known mol fraction of PS and the molar mass of PS unit.

^3^ *dn*/*dc* was determined according to (1).

**Table 2:** Molar-mass averages of the mixture of block copolymers determined by the 2D-LC and the calibration of SEC columns using PS standards.

Column	Spot 1 (copolymer 3)		Spot 2 (copolymers 1 and 2)	
	*M*_*n*_ (kDa)	*M*_*n*_ (kDa)	*M*_*w*_ (kDa)	*M*_*n*_ (kDa)
Nucleosil C18	71.2	66.4	38.1	35.7
Zorbax C8	67.0	64.7	39.5	37.1
